# Nicotinamide ameliorates mitochondria-related neuronal apoptosis and cognitive impairment via the NAD^+^/SIRT3 pathway

**DOI:** 10.1038/s41537-023-00357-w

**Published:** 2023-05-20

**Authors:** Keke Hao, Fashuai Chen, Linyao Zhao, Shilin Xu, Ying Xiong, Rui Xu, Xinhui Xie, Huan Huang, Chang Shu, Zhongchun Liu, Huiling Wang, Gaohua Wang

**Affiliations:** 1 Department of Psychiatry, Renmin Hospital of Wuhan University, 430060 Wuhan, China; 2 Department of Colorectal Surgery, The First Affiliated Hospital of Xinxiang Medical University, 453100 Henan, China; 3 Department of Neurosurgery, Renmin Hospital of Wuhan University, 430060 Wuhan, China; 4 Hubei Provincial Key Laboratory of Developmentally Originated Disease, 430071 Wuhan, China; 5 Hubei Institute of Neurology and Psychiatry Research, 430060 Wuhan, China

**Keywords:** Learning and memory, Schizophrenia

## Abstract

Emerging evidence suggests that mitochondria play a central role in mental health disorders including schizophrenia. Here we investigated whether nicotinamide (NAM) normalized cognitive impairment via a mechanism involving the mitochondrial Sirtuin 3 (SIRT3) pathway. The 24 h maternal separation (MS) rat model was used to mimic schizophrenia-associate phenotypes. Schizophrenia-like behaviors and memory impairments were detected using the pre-pulse inhibition test, novel object recognition test, and Barnes maze test, and neuronal apoptosis was characterized using multiple assays. SIRT3 activity was inhibited pharmacologically or by knockdown in HT22 cells, and BV2 microglia and SIRT3-knockdown HT22 cells were co-cultured in vitro. Mitochondrial molecules were measured by western blotting, and mitochondrial damage was measured with reactive oxygen species and mitochondrial membrane potential assays. Proinflammatory cytokines were assayed by ELISA and microglial activation was detected by immunofluorescence. MS animals showed behavioral and cognitive impairment and increased neuronal apoptosis. Supplementation with NAM or administration of honokiol, a SIRT3 activator, reversed all of the changes in behavioral and neuronal phenotypes. Administration of the SIRT3 inhibitor 3-TYP in control and NAM-treated MS rats caused behavioral and neuronal phenotypes similar to MS. In vitro, inhibition of SIRT3 activity with 3-TYP or by knockdown in HT22 cells increased ROS accumulation and caused neuronal apoptosis in a single-culture system. In co-culture systems, SIRT3 knockdown in HT22 cells activated BV2 microglia and increased levels of TNF-α, IL-6, and IL-1β. The administration of NAM blocked these alterations. Taken together, these data suggest that NAM can rescue neuronal apoptosis and microglial over-activation through the nicotinamide adenine dinucleotide (NAD^+^)-SIRT3-SOD2 signaling pathway, furthering our understanding of the pathogenesis of schizophrenia and providing avenues for novel treatments.

## Introduction

Schizophrenia is a devastating mental health disorder caused by complex genetic and environmental factors. Epidemiological studies have repeatedly shown that early life stress (ELS) such as abuse and neglect increase the risk of schizophrenia^1^. Cognitive impairments are considered a core symptom domain of schizophrenia^2^, and some studies have shown that neuronal apoptosis contributes to the development of this cognitive dysfunction^3,4^. However, the molecular mechanisms underlying neuronal apoptosis and cognitive deficits in schizophrenia are unclear.

ELS is thought to be a key susceptibility factor for psychotic diseases including schizophrenia, and several studies have confirmed that the abnormal behaviors and molecular changes taking place after a single 24 h period of maternal separation (MS) on postnatal day (PND) 9 in rats successfully recapitulate several features of schizophrenia^5–7^. Therefore, the MS rat model has become a powerful tool for exploring the neurobiological basis of schizophrenia.

Mitochondria are dynamic organelles in eukaryotic cells that play central roles in ATP production, cellular calcium buffering, and apoptosis^8^. Neurons are especially sensitive and vulnerable to abnormalities in mitochondrial function due to their high energy requirements^9^. Previous studies have reported that abnormal mitochondrial morphology caused by an impaired mitochondrial dynamics results in mitochondrial dysfunction, overproduction of reactive oxygen species (ROS), and subsequent apoptotic death of neurons^10,11^. Furthermore, decreased transcription of mitochondrial genes ultimately leads to decreased mitochondrial function (lower oxygen consumption rate, increased superoxide production) and increased apoptosis^12^.

Protein acetylation is a posttranslational process regulating global mitochondrial functions^13^. Sirtuin 3 (SIRT3) is a sirtuin family member located in the mitochondrial matrix that exhibits robust deacetylase activity^14,15^. SIRT3 regulates the activity of enzymes that regulate mitochondrial function such as manganese superoxide dismutase (MnSOD)^16^ and oligomycin sensitivity-conferring protein (OSCP)^17^ by deacetylation, thereby reducing the ROS overproduction under oxidative stress-dependent conditions such as aging and neural degeneration^14^. In addition, cellular nicotinamide adenine dinucleotide (NAD^+^) levels are a critical regulator of SIRT3 activity, and NAD^+^ depletion decreases SIRT3 activity and increases acetylation of its target proteins^18^. Our previous studies indicated that ELS such as MS can reduce NAD^+^ and SIRT3 expression in the CNS in rats and that nicotinamide (NAM) administration can ameliorate these biochemical changes as well as some ELS-induced cognitive deficits^19^. However, SIRT3’s involvement in the neuronal impairment induced by ELS and whether it is key to the neuroprotective effects of NAM in this model of schizophrenia are uncertain.

Therefore, the purpose of this study was to explore whether the neuroprotective effect of NAM is mainly regulated by a mechanism involving the NAD^+^-SIRT3-SOD mitochondrial pathway in vitro and in vivo.

## Materials and methods

### Animals and maternal separation

Forty nulliparous female and 40 male eight-week-old Wistar rats were purchased from Beijing Vital Rival Laboratory Animal Technology Co., Ltd. (Beijing, China). Three to four rats of the same sex were caged together. Animals were mated at three months of age and the males removed one week later. The mated female rats were housed individually in ventilated plastic cages in a temperature- and humidity-controlled (22 ± 20 °C, 50 ± 10%) holding facility with a constant 12 h day-night cycle (lights: 08:00–20:00). All animals had free access to food and tap water.

MS was performed as previously^6,20^. Females were checked twice daily for delivery (08:00 and 17:00), and the day of delivery was considered post-natal day (PND)0. Each pregnant rat provided on average 10 ± 2 offspring. On PND9, about twenty rat litters were randomly subjected to MS for 24 h. In brief, for MS, the mothers were removed at 10:00. The pups remained in their home cages at room temperature for 24 h. On PND10, the mothers were placed back in their cages. The control groups grew naturally without MS to adulthood. All litters were otherwise left undisturbed except for the routine cleaning of cages. On PND21, the MS and control pups were weaned and then group-housed by sex (3–4 per cage). Since previous studies have shown that estrogen plays a key role in regulating neuronal activity and animal behavior, and alterations in estrogen signaling are linked to a range of neurological and psychiatric conditions^21,22^, all subsequent experiments were carried out only on male offspring^23^. All procedures involving animals were approved and carried out according to the guidelines of the Institutional Animals Care Committee of Renmin.

### Experimental design

*Experiment 1:* On PND9, the dams with their pups were randomly divided into a control group and an MS group. On PND10 after MS, each infant group was randomly divided into several subgroups. The resulting four groups (PND56) were: control and saline group (Control+Saline), MS and saline group (MS + Saline), control and NAM group (Control + NAM); and MS and NAM group (MS + NAM). The NAM groups were gavaged with NAM (100 mg/kg/d, diluted in saline; Sigma-Aldrich, St Louis, MO, USA; catalog no. N0636) for 30 days from PND56 to PND85. The NAM dosage and treatment protocol were as previously^24,25^. The saline groups received saline (1 ml/kg/d) from PND56 to PND85 as vehicle control.

*Experiment 2:* About 60 infant pups in the MS group and the control group were randomly divided into two subgroups of 15 each and were intraperitoneally injected with 3-TYP (a SIRT3 inhibitor; 10 mg/kg/d; Cayman Chemical Company, Ann Arbor, MI; CAS: 120241-79-4)^26^, honokiol (HNK) (a SIRT3 activator, 10 mg/kg/d; Cayman Chemical Company CAS: 35354-74-6)^27^, or saline respectively for 15 days from PND56 to PND70. The resulting four groups were: Control+Saline group (Control+Saline); MS + Saline group (MS + Saline); MS and HNK group (MS + HNK); and Control and 3-TYP group (Control+3-TYP). The saline groups received a saline intraperitoneal injection (1 ml/kg/d).

*Experiment 3:* The remaining 36 MS rats were divided into MS + Saline, MS + NAM, and MS + NAM + 3-TYP groups. The MS + NAM + 3-TYP group was gavaged with NAM for 45 days from PND56 to PND100 and intraperitoneally injected with 3-TYP (10 mg/kg/d) over the last 15 days from PND86 to PND100. The MS + NAM group was gavaged with NAM for 45 days from PND56 to PND100. The MS + Saline group was gavaged with saline (1 ml/kg/d) for 45 days from PND56 to PND100 and intraperitoneally injected with saline (1 ml/kg/d) for the last 15 days from PND86 to PND100.

### Behavioral testing of animals

Adult experimental rats were evaluated using the novel object recognition test, the Barnes maze test, and pthe re-pulse inhibition (PPI) test. The novel object recognition test was completed during the first three days. A standard Barnes maze test behavioral protocol was completed between the 2nd and 5th days to evaluate cognition, including working and spatial recognition memory. On the last day, a pre-pulse inhibition (PPI) test was completed to measure sensorimotor gating. All behavioral tests were performed between 08:00 and 22:00. Each rat was assessed for each behavioral measure. After each trial, the apparatus was cleaned with 75% alcohol.

#### Novel object recognition test

The novel object recognition test was performed using the open-field apparatus and had three components: habituation, training, and testing^28^. Rats were placed in the center of the open field to adapt to the apparatus for 24 h. During the training experiment, rats were allowed to explore two identical objects for 10 min. The test experiment started 24 h later. Rats were allowed to explore one familiar and one novel object (replacing a training object) for 10 min, with locomotion tracked by video and analyzed using Panlab SMART v3.0 (Barcelona, Spain). The formula for calculating preference for exploring the novel object was: [(time spent in the novel subject) / (total time spent in two subjects) × 100%].

#### Barnes maze test

We examined spatial memory with the Barnes maze^29^, which was elevated 140 cm above the floor and consisted of 20 holes located evenly on the surface periphery, each 10 cm in diameter. The target box was a hole that connected to a dark chamber, allowing the animal to escape from bright light exposure. The day before the formal experiment, animals were adapted to the target box for 4 min. On the first day, each animal was placed in the center black cube of the maze for 5 s and permitted to explore the maze to find the target box when the cube was removed. Once the animal entered the escape box, it was left there for 30 s; if it failed to find the target box within 3 min, it was taken to the target box and allowed to remain in the target box for 1 min. Each animal underwent two trials during the day with an interval of 4 h. The latency time to reach the target box was recorded. The test was performed over four days. The whole process was monitored by a digital camera and a computer system.

#### PPI test

PPI testing was conducted in a sound-attenuated chamber equipped with a small plexiglas cage mounted on a load-cell platform to digitize the pressure generated by the startled rat (AniLab Scientific Instruments Co., Ltd., China; www.anilab.cn). This test was performed with a broad-band white noise of 70 dB. A high-pass (>4 kHz) white noise was used as the pulse stimulus. Following acclimatization for 5 min, a sequence of five startle stimuli (120 dB of startle stimulus for 20 ms) was applied. During the testing phase, the initial delay was 50 ms followed by a 20 ms impulse stimulus (75, 78, or 82 dB) and then a 100 ms delay followed by a 40 ms startle stimulus of 120 dB randomly applied for about 40 trials by software control. The interstimulus interval ranged from 10 to 30 s. The formula for calculating the percentage PPI caused by the intensity of each pre-pulse was [1-(startle amplitude on pre-pulse trial)/(startle amplitude on pulse alone)] × 100%^30^.

### Sample collection

After completing behavioral testing, the animals were euthanized using sodium thiopental (50 mg/kg, i.p.). From about 15 animals per group, brain tissues were taken and frozen immediately on dry ice and then transferred to −80 °C until required for further dissection and analysis for gene and protein expression.

### Cell culture

Mouse hippocampal neuron HT22 cells were obtained from the cell bank of the Shanghai Institute for Biological Sciences, Chinese Academy of Sciences (Shanghai, China). Cells were cultured in high-glucose DMEM (Gibco, Thermo Fisher Scientific, Inc., Waltham, MA, USA) supplemented with 10% FBS, 1% streptomycin, and 1% penicillin (Beijing Solar Science & Technology Co., Ltd., China) at 37 °C with 5% CO_2_ in a humid atmosphere. Cells were seeded in a six-well plate at a density of 2 × 10^5^ cells/well followed by treatment with or without a 50 μM 3-TYP and 5 mM NAM for 24 h.

### Construction and transfection of shRNA targeting SIRT3

An shRNA targeting SIRT3 (shSIRT3) was designed and synthesized using the Miaoling Plasmid Platform (http://www.miaolingbio.com/). HT22 cells were transfected using Lipofectamine 3000 (Thermo Fisher Scientific) according to the manufacturer’s instructions. Knockdown was verified by western blotting.

### Co-cultures

HT22 cells were seeded into six-well plates. The transfection of shSIRT3 and shNC (shRNA negative control) was conducted at approximately 60% confluence. Approximately 2 × 10^5^ BV2 microglial cells were seeded in each well 36 h after transfection followed by treatment with or without 5 mM NAM for 24 h.

### TUNEL staining

Four rats from each group were anesthetized and perfused through the heart initially with PBS followed by 4% paraformaldehyde. The brain was then post-fixed in 4% paraformaldehyde overnight. Brains were embedded in paraffin and 4 μm sections cut. Paraffin-embedded tissue cross-sections were dewaxed in xylol, rehydrated, placed in sodium citrate, microwaved for antigen retrieval, and then washed with PBS and blocked with 1% BSA (Roche, Basel, Switzerland) in PBS for 2 h at room temperature. Sections were incubated at 4 °C overnight with primary antibodies targeting mouse NeuN (1:100, ab279296, Abcam, Cambridge UK), followed by incubation with an Alexa Fluor FITC-conjugated goat anti-mouse IgG secondary antibody (1:250, Bost Biotech, China) for 60 min. Three tissue sections were selected from each animal for staining and counting of positive cells, with the average value taken as the positive cell count for that animal.

Cells were fixed in 4% paraformaldehyde for 30 min and incubated with TUNEL reaction mixture for 1–1.5 h. Next, sections were developed with peroxidase solution and diaminobenzidine, counterstained with hematoxylin, dehydrated in gradient alcohol, permeabilized, and sealed in neutral gum. DAPI (Thermo Fisher Scientific) was used as a nuclear stain. Images were obtained using an inverted fluorescence microscope (Olympus BX51; Olympus, Tokyo, Japan). Image J (National Institutes of Health, Bethesda, MD) was used to analyze the integral optical density (IOD) of the target protein.

### Protein extraction and western blot analysis

Total protein was extracted from cultured HT22 cells using protein lysis buffer (Beyotime Biotech, China). The protein concentration was determined with a BCA kit (P0010S, Beyotime Biotech). The proteins in each sample were separated by 10% SDS-polyacrylamide gel electrophoresis and transferred onto polyvinylidene fluoride (PVDF) membranes. Western blot analysis was carried out using the following primary antibodies raised against target proteins: rabbit anti-SIRT3 (dilution 1:1000, ab189860, Abcam), rabbit anti-SOD2 (dilution 1:1000, ab68155, Abcam), rabbit anti-acetyl-SOD2 (dilution 1:1000, AF3751, Affinity Biosciences, Melbourne, Victoria, Australia), rabbit anti-cleaved caspase 3 (dilution 1:1000, 19677-1-AP, Proteintech, Fisher Scientific), and rabbit anti-β-actin (dilution 1:1000, ab181602, Abcam) antibodies overnight at 4 °C. The following secondary horseradish peroxidase (HRP)-conjugated antibodies were used at 1:5000 dilution: goat anti-rabbit HRP (Millipore, Watford, UK). Blots were visualized with enhanced chemiluminescence detection reagents using a Chemidoc TM Touch Imaging System (Bio-Rad, Hercules, CA).

### Detection of intracellular ROS

ROS generation was detected using a ROS assay kit (cat. no. S0033S; Beyotime Biotech) according to the manufacturer’s protocol. 10 µM 2,7-dichloride-hydrofluorescein diacetate (DCFH-DA) was added to each well and incubated at 37 °C for 30 min in the dark. The fluorescence intensity was examined with a microscope to evaluate ROS generation. ImageJ software was used to measure the fluorescence intensity.

### Detection of mitochondrial membrane potential (MMP)

The collapse of MMP is a hallmark event of early apoptosis. Changes in MMP were evaluated by JC-1 staining according to the manufacturer’s instructions (MMP assay kit with JC-1; no. C2006; Beyotime Biotech).

### Apoptosis assay

An Annexin V/PI kit (BD Biosciences, Franklin Lakes, NJ, USA) was used to measure microglial cell apoptosis. Cells were seeded in six-well plates and transfected with the corresponding shRNAs or treated with 3-TYP or NAM. According to the manufacturer’s instructions, fixed cells were suspended in 1 ml 1X binding buffer and stained with Annexin V-PE/7-ADD for 10 min in the dark. For each experiment, 2 × 10^5^ cells were analyzed with a FACSCalibur flow cytometer (BD Biosciences). Early apoptosis and late apoptosis were summed to calculate the total apoptosis rate.

### Immunofluorescence, imaging, and quantification in co-cultures

Co-cultures were fixed in 4% paraformaldehyde for 30 min after adding BV2 cells for 24 h. Cells were blocked with 5% bovine serum albumin for 1 h. Co-cultures were incubated with anti-CD68 (1:100, ab125212, Abcam) overnight at 4 °C followed by Alexa-CY3 secondary fluorescent antibodies (1:400, Invitrogen) at 37 °C for an hour. DAPI was used as a nuclear stain. Slides were imaged with a microscope (Olympus BX51). The images were analyzed individually to evaluate CD68 expression, and the immunofluorescence intensity of CD68 per field was determined using ImageJ software.

### Enzyme-linked immunosorbent assay (ELISA)

Cell culture supernatants were carefully collected after co-culture for 24 h and centrifuged at 4 °C at 12,000 × *r* for 15 min. Cytokine concentrations were evaluated using protein assay kits (Mouse TNF-α ELISA Kit, Abcam, ab208348; Mouse IL-6 ELISA Kit, Abcam, ab222503; Mouse IL-1β ELISA Kit, Abcam, ab197742) following the manufacturer’s guidelines. Cell cytokine concentrations are expressed as picograms per milliliter.

### Data analysis and statistics

Data are presented as means ± standard error of the mean (SEM) and were analyzed with SPSS Statistics v20.0 (IBM Statistics Inc., Armonk, NY, USA). Data were analyzed by analysis of variance (ANOVA) to determine group differences. A probability level of less than 0.05 was accepted as significant.

## Results

### MS rats show cognitive impairment and NAM administration normalizes behavior

**Fig. 1:**
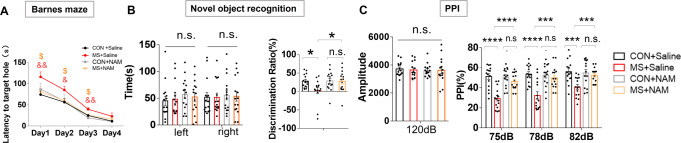
NAM administration attenuates PPI and cognitive deficits induced by MS. **A** Effects of NAM administration on the latency to the target hole in the Barnes maze test. ^&^*p* < 0.05, ^&&^*p* < 0.01 as compared with MS + Saline rats, ^#^*p* < 0.05, ^##^*p* < 0.05 as compared with MS + NAM rats. **B** Effects of NAM administration on the novel object recognition assay in different groups. **C** Effects of NAM administration (100 mg/kg) on PPI at different pre-pulse intensities. CON + Saline, *n* = 15; MS + Saline, *n* = 15; CON + NAM, *n* = 15; MS + NAM, *n* = 15. n.s.—not significant; *p < 0.05 and **p < 0.01 as compared with controls. Data are represented as mean ± SEM.

To investigate the effect of MS on rat behaviors, we first performed several behavioral tests to establish baselines. In the Barnes maze test, MS rats showed deficiencies in spatial learning and memory. Compared with controls, MS rats showed a greater latency to escape from day 1 to day 3 (Fig. 1A). The MS + NAM group showed decreased latency entering the target hole compared with the MS + Saline group (Fig. 1A).

We next assessed the cognitive function of MS rats using the novel object recognition test. The time taken to explore the two identical objects in the adaptation stage was similar between the four groups (Fig. 1B, left panel). However, the MS group spent relatively less time exploring the novel object when one of the objects was replaced after 24 h (Fig. 1B, right panel), while the MS + NAM group spent more time sniffing the novel object than the MS + Saline group (Fig. 1B, right panel).

Finally, in the PPI test, there was no difference in baseline startle reactivity between groups (Fig. 1C, left panel). MS rats exhibited impaired PPIs with varying degrees of pre-pulsing, indicating reduced inhibition of the startle stimulus compared with control rats. The impaired PPI of the MS group was ameliorated to some extent after the rats were administered 100 mg/kg NAM (Fig. 1C, right panel).

### NAM decreases neuronal apoptosis in MS rats

**Fig. 2:**
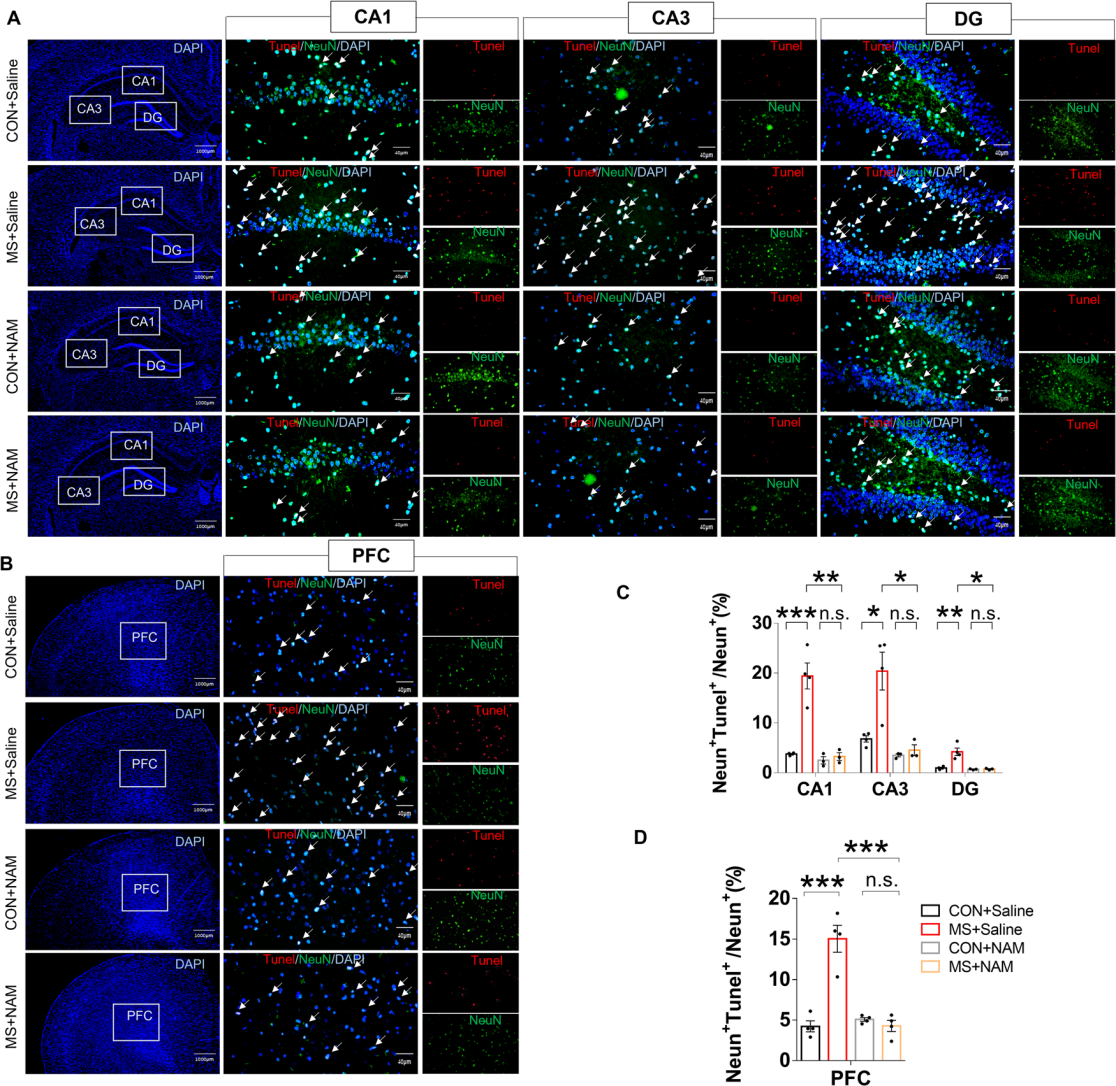
NAM administration reverses the effects of MS on neuronal apoptosis in the hippocampus and PFC. **A** Neuronal apoptosis was assessed by TUNEL staining in the hippocampus. TUNEL positive cells (red), NeuN (green), and DAPI (blue) labeling. **B** Neuronal apoptosis was assessed by TUNEL staining in the PFC. TUNEL positive cells (red), NeuN (green), and DAPI (blue) labeling. **C** Quantification of TUNEL‐positive cells in the hippocampus (*n* = 4 per group). **D** Quantification of TUNEL‐positive cells in the PFC (*n* = 4 per group). Data are presented as mean ± SEM for each group. n.s.—not significant; *p < 0.05.

Neuronal apoptosis was assessed by TUNEL staining. The number of TUNEL‐positive cells increased in the MS group in the hippocampal CA1, CA3, and dentate gyrus regions (Fig. 2A, B) and in the prefrontal cortex (PFC; Fig. 2A, C) compared with the control group. NAM administration in the MS + NAM group decreased neuronal apoptosis in the hippocampus (Fig. 2A, B) and PFC (Fig. 2A, C) compared with the MS + Saline group.

### SIRT3 mediates the effect of NAM to normalize neuronal and behavioral phenotypes

**Fig. 3:**
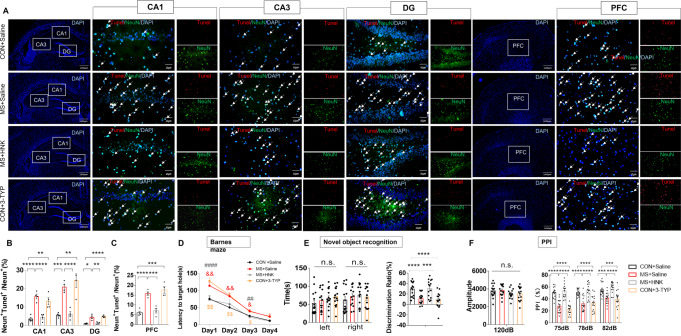
Effects of activation and inhibition of SIRT3 on neuronal apoptosis and behavior phenotypes. **A** Neuronal apoptosis was assessed by TUNEL staining in the hippocampus and PFC. TUNEL positive cells (red), NeuN (green), and DAPI (blue) labeling. **B** Quantification of TUNEL‐positive cells in the hippocampus (*n* = 4 per group). **C** Quantification of TUNEL‐positive cells in the PFC (*n* = 4 per group). **D** Effects of HNK and 3-TYP administration on the latency to the target hole in the Barnes maze test (*n* = 15, per group). ^&^*p* < 0.05, ^&&^*p* < 0.01 as compared with MS + Saline rats, ^##^*p* < 0.01, and ^####^*p* < 0.001 as compared with MS + HNK rats. ^$^*p* < 0.05, and ^$$^*p* < 0.01 as compared with CON + 3-TYP rats. **E** Effects of HNK and 3-TYP administration on the novel object recognition assay in different groups (*n* = 15, per group). **F** Effects of HNK and 3-TYP administration on PPI at different pre-pulse intensities (*n* = 15, per group). n.s. was not significant; *p < 0.05, **p < 0.01, ***p < 0.001, and ****p < 0.0001. The data are represented as the mean ± SEM.

Compared with MS rats, TUNEL analysis revealed that the number of TUNEL‐positive cells decreased after 15 days of HNK administration in MS + HNK rats in the hippocampus (Fig. 3A, B) and PFC (Fig. 3A, C), and the number of TUNEL‐positive cells increased in the Control+3-TYP rats compared with the Control+Saline rats in the hippocampus (Fig. 3A, B) and PFC (Fig. 3A, C).

Furthermore, in the Barnes maze test, MS + HNK rats showed decreased latency entering the target hole compared with MS + Saline rats (Fig. 3D). Compared with the Control+Saline group, Control+3-TYP rats showed prolonged latency to escape (Fig. 3D). In the novel object recognition test, the four groups showed no significant difference in the time exploring the two identical objects in the adaptation stage (Fig. 3E, left panel). The MS + HNK group spent more time sniffing the novel object after 24 h than the MS + Saline group (Fig. 3E, right panel). However, Control+3-TYP rats spent relatively less time exploring the novel object when one of the objects was replaced after 24 h compared with Control+Saline rats (Fig. 3E, right panel). In the PPI test, there was no difference between groups in baseline startle reactivity (Fig. 3F, left panel). The impaired PPI of the MS + Saline group was to some extent ameliorated after rats were administrated HNK (Fig. 3F, right panel). Control+3-TYP rats exhibited impaired PPIs at varying degrees of pre-pulsing, indicating reduced inhibition of the startle stimulus compared with Control+Saline rats (Fig. 3F).

### SIRT3 inhibition blocks NAM-induced rescue of the neuronal and behavioral phenotype

**Fig. 4:**
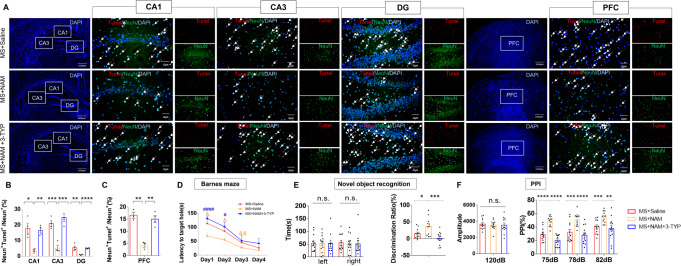
SIRT3 inhibition blocks NAM-induced rescue of neuronal apoptosis and behavioral phenotypes. **A** Neuronal apoptosis was assessed by TUNEL staining in the hippocampus and PFC. TUNEL positive cells (red), NeuN (green), and DAPI (blue) labeling. **B** Quantification of TUNEL‐positive cells in the hippocampus (*n* = 4 per group). **C** Quantification of TUNEL‐positive cells in the PFC (*n* = 4 per group). **D** 3-TYP administration blocked NAM-induced rescue of the latency to the target hole in the Barnes maze test (*n* = 12, per group). ^&^*p* < 0.05, ^&&^*p* < 0.01 as compared with MS + NAM rats. ^#^*p* < 0.05, and ^####^*p* < 0.0001 as compared with MS + NAM + 3-TYP rats. **E** 3-TYP administration blocked NAM-induced rescue in the novel object recognition assay in different groups (*n* = 12, per group). **F** 3-TYP administration blocked NAM-induced rescue on PPI at different pre-pulse intensities (*n* = 12, per group). n.s. was not significant; *p < 0.05, **p < 0.01, ***p < 0.001, and ****p < 0.0001. The data are represented as the mean ± SEM.

3-TYP administration in the MS + NAM + 3-TYP group blocked the NAM-induced decrease in the number of TUNEL‐positive cells in the hippocampus (Fig. 4A, B) and the PFC (Fig. 4A, C).

Furthermore, 3-TYP administration significantly blocked the recovery of cognitive impairment in MS + NAM rats. In the Barnes maze test, MS + NAM + 3-TYP rats showed a greater latency to escape to the target hole from day 1 to day 2 compared with the MS + NAM rats (Fig. 4D). Novel object recognition testing revealed that MS + NAM + 3-TYP rats spent relatively less time exploring the novel object when one of the objects was replaced after 24 h compared with MS + NAM rats (Fig. 4E, right panel). In the PPI test, there was no difference in baseline startle reactivity between groups (Fig. 4F, left panel). MS + NAM + 3-TYP rats exhibited impaired PPIs with varying degrees of pre-pulsing (Fig. 4F, right panel).

### SIRT3 inhibition causes mitochondrial damage and increases apoptosis in HT22 cells

**Fig. 5:**
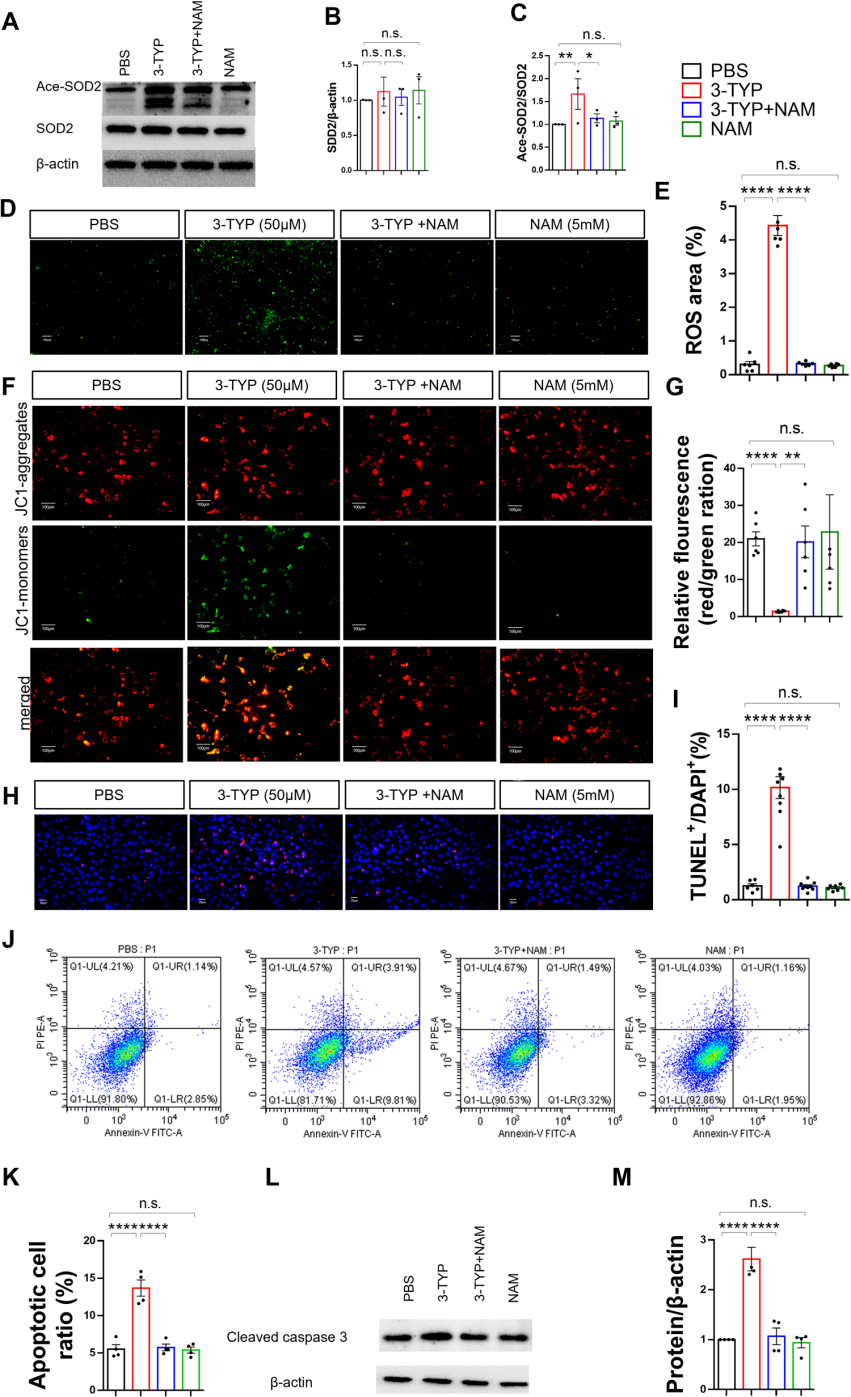
SIRT3 inhibition causes mitochondrial damage and increases apoptosis in HT22 cells. **A**–**C** Immunoblots and quantification analysis of the level of acetylated SOD2 in HT22 cells (*n* = 3, per group). Data were normalized to controls. **D** Representative images of ROS (green) levels in HT22 cells. **E** Quantitative analyses of the percentage ROS area the HT22 cells (*n* = 6, per group). **F** Representative images of the mitochondrial membrane potential in HT22 cells. **G** Bar graph presenting the green/red fluorescence ratio, which reflects changes in the mitochondrial membrane potential. **H** Neuronal apoptosis was assessed by TUNEL staining in HT22 cells. TUNEL positive cells (red), DAPI (blue) labeling. **I** Quantification of TUNEL‐positive cells in HT22 cells (*n* = 4 per group). **J** Apoptotic HT22 cells were detected by flow cytometry. **K** The percentage of apoptotic cells was determined (*n* = 4, per group). **L**, **M** Immunoblots and quantification analysis of cleaved caspase 3 levels in HT22 cells (*n* = 4, per group). Data were normalized to controls. The data are presented as mean ± SEM for each group. n.s. was not significant; *p < 0.05, **p < 0.01, and ****p < 0.0001.

Compared with culture in PBS alone, 3-TYP increased SOD2 acetylation (Fig. 5A–C) and ROS levels (Fig. 5D, E) in HT22 cells. Mitochondrial membrane potential (MMP) assays indicated an increased green/red fluorescence ratio and decreased MMP in HT22 cells after treatment with 3-TYP (Fig. 5F, G). Furthermore, compared with PBS culture, 3-TYP increased apoptosis in HT22 cells as assessed by TUNEL (Fig. 5H, I) after 3-TYP administration for 24 h, a result confirmed by flow cytometry analysis (Fig. 5J, K). Western blotting also revealed that cleaved caspase 3 expression significantly increased after 3-TYP administration (Fig. 5L, M), with NAM administration blocking these effects (Fig. 5A–M).

### SIRT3 knockdown damages mitochondria and increases apoptosis in HT22 cells

**Fig. 6:**
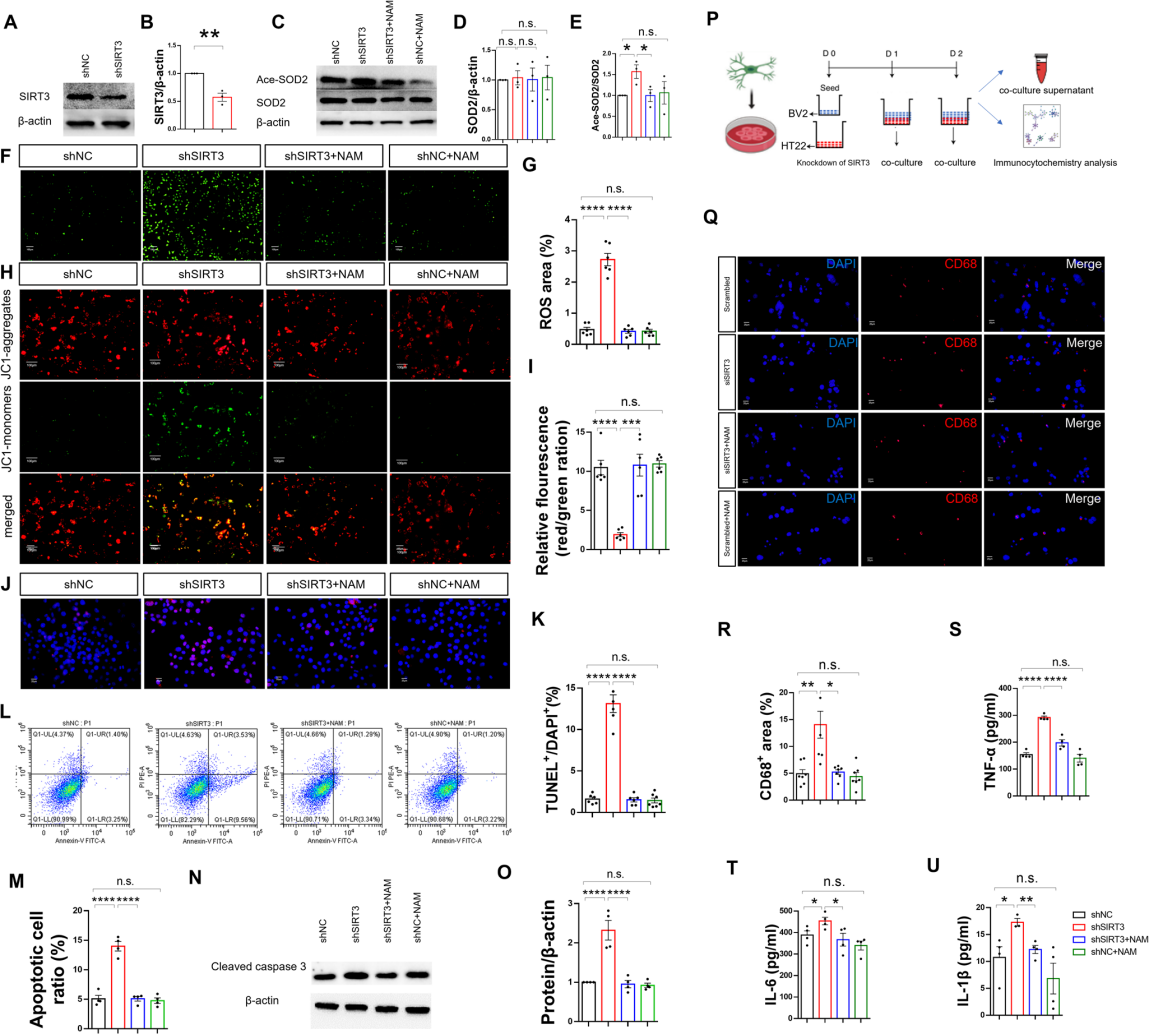
SIRT3 knockdown causes mitochondrial damage in HT22 cells and induces microglial activation. **A** Immunoblots of the SIRT3 knockdown effect in HT22 cells by western blotting. **B** Quantification analysis of SIRT3 knockdown in HT22 cells (*n* = 3, per group). Data were normalized to controls. **C**–**E** Immunoblots and quantification analysis of acetylated SOD2 levels in HT22 cells (*n* = 3, per group). Data were normalized to controls. **F** Representative images of ROS (green) levels in the HT22 cells. **G** Quantitative analyses of the percentage ROS area in HT22 cells (*n* = 6, per group). **H** Representative images of the mitochondrial membrane potential in HT22 cells. **I** Bar graph presenting the green/red fluorescence ratio, which reflects changes in the mitochondrial membrane potential. **J** Neuronal apoptosis assessed by TUNEL staining in HT22 cells. TUNEL positive cells (red), DAPI (blue) labeling. **K** Quantification of TUNEL‐positive cells in HT22 cells (*n* = 4 per group). **L** Apoptotic HT22 cells were detected by flow cytometry. **M** The percentage of apoptotic cells was determined (*n* = 4, per group). **N**, **O** Immunoblots and quantification analysis of the cleaved caspase 3 levels in HT22 cells (*n* = 4, per group). Data were normalized to controls. **P** Scheme for co-culture of shSIRT3 HT22 cells with BV2 cells. **Q** Representative images of CD68-positive cells in co-cultures of shSIRT3 HT22 cells with BV2 cells. CD68-positive cells (red), DAPI (blue) labeling. **R** Quantification of CD68-positive cells in co-cultures of shSIRT3 HT22 cells with BV2 cells (*n* = 7, per group). **S** Secretion of TNF-α into co-culture supernatants was measured by ELISA (*n* = 4, per group). **T** Secretion of IL-6 into co-culture supernatants was measured by ELISA (*n* = 4, per group). **U** Secretion of IL-1β into co-culture supernatants was measured by ELISA (*n* = 4, per group). Data are presented as mean ± SEM for each group. n.s. was not significant; *p < 0.05, **p < 0.01, ***p < 0.001, and ****p < 0.0001.

Compared with negative controls, shSIRT3 decreased SIRT3 and increased SOD2 acetylation (Fig. 6A–E) and ROS levels (Fig. 6F, G) in HT22 cells. MMP assays indicated an increased green/red fluorescence ratio and decreased MMP in shSIRT3-treated cells (Fig. 6H, I). Furthermore, compared with the negative control group, there was increased apoptosis in the 3-TYP shSIRT3 group by TUNEL (Fig. 6J, K). By flow cytometry, apoptotic cells increased after SIRT3 knockdown (Fig. 6L, M) and, consistent with this, western blotting revealed significantly increased cleaved caspase 3 expression on SIRT3 knockdown (Fig. 6N, O). NAM administration rescued the apoptotic phenotype (Fig. 6A–O).

### shSIRT3 HT22 cells activate co-cultured BV2 microglial cells and influence secretion of pro-inflammatory cytokines

To further characterize responses of HT22 cells on *SIRT3* knockdown, we analyzed engulfment of BV2 microglial cells in co-culture experiments. CD68 levels significantly increased in BV2 cells exposed to shSIRT3 HT22 cells (Fig. 6Q, R) and, furthermore, BV2 cells co-cultured with shSIRT3 HT22 cells induced secretion of TNF-α, IL-6, and IL-1β (Fig. 6S–U). The co-incubation of NAM with activated BV2 cells significantly diminished the secretion of the pro-inflammatory cytokines TNF-α, IL-6, and IL-1β and CD68 expression (Fig. 6Q–U).

## Discussion

**Fig. 7:**
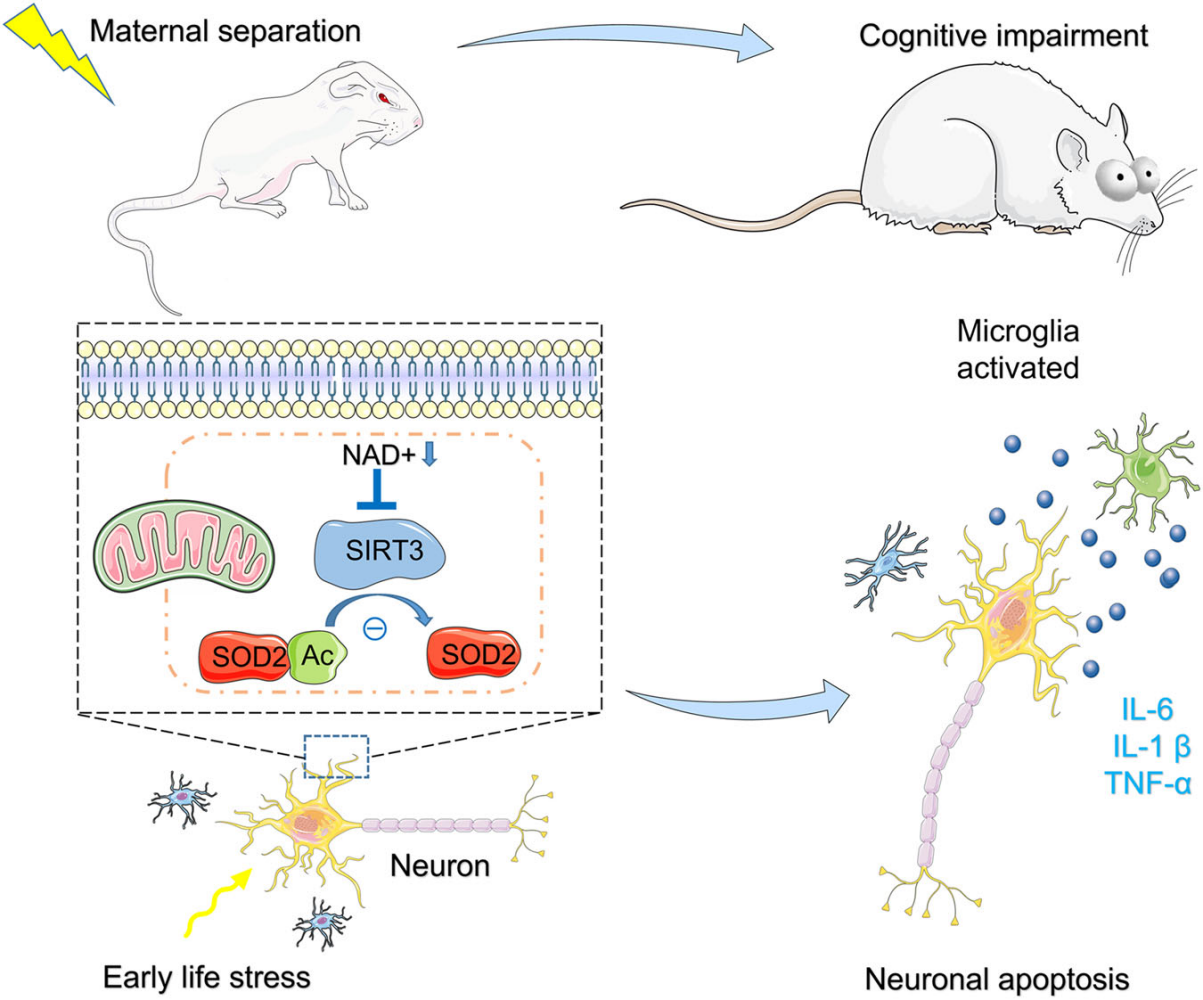
A schematic illustrating how SIRT3 might mediate the stabilizing effect of NAD^+^ on normalized cellular and behavioral phenotypes. Maternal separation, as an early life stress, decreases NAD^+^ and SIRT3 levels, induces functional abnormalities in mitochondria, activates microglia, increases neuronal apoptosis in the hippocampus and prefrontal cortex, and induces cognitive deficits. SIRT3 mediates the stabilizing effect of NAD^+^ on normalized cellular and behavioral phenotypes.

We previously observed that MS, as an early life stress, induces long-term cognitive deficits, behavioral changes, and an increase in neuronal apoptosis in the CNS^19^. We now show that NAM treatment recovers MS-induced neuronal and behavioral phenotypes in adult rats. Treatment with HNK (a SIRT3 activator) similarly relieved the cognitive deficits and neuronal apoptosis induced by MS, while 3-TYP (a SIRT3 inhibitor) induced neuronal apoptosis and cognitive deficits in control and NAM-treated adult MS rats. In vitro, inhibition of SIRT3 activity with 3-TYP or *SIRT3* knockdown in HT22 cells increased SOD2 acetylation, ROS, and neuronal apoptosis in a single-culture system. In BV2 and HT22 co-cultures, SIRT3 knockdown in HT22 cells induced microglial over-activation, and NAM administration rescued neuronal apoptosis and microglial cell over-activation by regulating the SIRT3-SOD2-ROS signaling pathway (Fig. 7).

A striking aspect of this study was our observation of the regulation of the mitochondrial molecules NAD^+^/SIRT3 in an animal model of schizophrenia-related behavioral and neuronal impairment together with a mechanistic dissection of the neurobiological intermediaries in both cells and animals. Mitochondrial dysfunction has frequently been reported in schizophrenia^31,32^. ELS leads to myriad behavioral changes^5–7,20,33^ and alters mitochondria gene expression and metabolism in different brain regions^34,35^. Indeed, a variety of antioxidants such as resveratrol^36,37^, curcumin^38^, and idebenone have been shown to improve cognitive deficits^39,40^. However, the causal implications of specific mitochondrial dysfunctions in stress-induced behavioral changes are unknown. MS is a well-established and validated rodent model of schizophrenia-related behaviors and cognitive deficits in adulthood^20,41,42^. In line with these studies, we found that PND9 MS rats spent less time exploring the novel object in the novel object recognition test and showed a greater latency to escape from day 1 to day 3 in the Barnes maze test. Additionally, PPI, which measures sensorimotor gating after the startle response, was also disrupted. Although the underlying neural mechanisms remain elusive, PPI impairments are present in several neuropsychiatric disorders, including schizophrenia^43,44^, and are studied as a behavioral assay in animal models of schizophrenia^45,46^. NAM (a precursor of NAD^+^) administration normalized impaired sensorimotor gating and cognitive deficits caused by MS. In addition, the SIRT3 activator HNK attenuated most abnormal behaviors caused by MS. Meanwhile, the SIRT3 inhibitor TYP led to abnormal PPIs and impaired cognitive behaviors in adult control and NAM-treated MS rats. Therefore, a distinct and novel contribution of this study was the identification of a key role for the mitochondrial molecule SIRT3 in the regulation of ELS-induced impairments in cognitive behavior.

Neuronal apoptosis is strongly associated with neurocognitive function^47^. Structural and functional abnormalities of mitochondria are closely related to apoptosis^48^. SIRT3, as the main mitochondrial sirtuin, regulates oxidative stress^49,50^ and is involved in protecting stress-induced mitochondrial integrity and energy metabolism^14^. Here, MS rats showed increased neuronal apoptosis in the hippocampus and PFC. Administration of HNK, a SIRT3 activator, to MS rats blocked pathological behavioral and neuronal phenotypes. Additionally, treating normal rats with 3-TYP (a SIRT3 inhibitor) caused cognitive impairment and increased neuronal apoptosis in the hippocampus and PFC. These findings are consistent with a previous study indicating that activated SIRT3 effectively ameliorated surgery/anesthesia-induced cognitive decline and neuronal apoptosis in mice^51^.

In vitro experiments revealed that knockdown of SIRT3 expression in HT22 cells damaged mitochondria and induced apoptosis. SOD2 acetylation, ROS, and apoptosis also increased in HT22 cells treated with a SIRT3 inhibitor. A previous study suggested that SIRT3 deficiency contributes to oxidative stress-induced melanocyte apoptosis via exacerbation of mitochondrial damage and cytochrome c release into the cytoplasm to activate apoptotic pathways^52^, supporting our results. Furthermore, NAM blocked these negative processes in HT22 cells treated with 3-TYP, while 3-TYP eradicated cognitive recovery and neuroprotection of the hippocampus and PFC in NAM-induced MS rats. These findings suggest that the neuroprotective effects of NAM, at least in part, involved activation of the SIRT3 signaling pathway.

To further explore the mechanism of cognitive impairment caused by neuronal injury through SIRT3 signaling, we also performed a series of in vitro experiments. shSIRT3 knockdown resulted in obvious neuronal damage in a single-culture system. Furthermore, we co-cultured HT22 cells with BV2 cells and found that unilateral knockdown of SIRT3 expression in HT22 cells activated BV2 microglia. As the principal cells involved in the innate immune response in the CNS, microglia can be activated by exogenous or endogenous ligands and produce several proinflammatory cytokines implicated in neurotoxicity. Numerous studies have shown that ELS can activate the neuroimmune system and alter the pro-inflammatory state of the CNS, causing abnormal development of critical brain areas, which drives the pathogenesis of psychiatric illness^53,54^. A previous study suggested that proinflammatory cytokine (IL-1β, IL-6, IL-8, and TNFα) release was enhanced in whole blood from schizophrenia patients^55^. Postmortem studies have also shown inflammatory markers in the dorsolateral PFC, and microglial activity and microglial cellular density were all increased in schizophrenia patients^56–59^. Our previous study showed that MS led to microgliosis and simultaneously caused microglial activation in the CNS of adult rats, and mRNA levels of TNF-α, IL-1β, and IL-6 increased in the hippocampus^19^. The view that over-activated microglia-mediated neuroinflammation contributes to neurotoxicity in the developing brain is supported by both in vitro and in vivo data^60–62^. Some studies have also reported that the damage was more severe in neurons co-cultured with activated microglia^63^. Several studies have reported that excessive microglial activation in the CNS increases neuronal apoptosis^64,65^. Thus, activated microglia clearly play a critical role in neuronal damage. Avoiding or reducing microglial activation and alleviating an inflammatory response would be an effective strategy to prevent or treat mental illnesses like schizophrenia.

Overall, our study demonstrated that the effect of low expression of SIRT3 on neuronal damage in MS rat models is multifaceted. On the one hand, it can cause mitochondrial damage directly by promoting neuronal apoptosis. On the other, it might activate microglia to aggravate neuronal injury. Our study also suggests that increasing systemic SIRT3 levels may have a neuroprotective effect. Previous studies have reported that, in the mitochondrial respiratory chain, CoQn (ubiquinones) oxidizes NADH to NAD^+^ in complex I (NADH-Q oxidoreductase), and increased NADH in the mitochondrial respiratory chain could increase respiration and decrease ROS production^66,67^. Furthermore, synaptic dysfunction has frequently been reported in schizophrenia^68,69^. Future studies should investigate the contribution of NAM to neuronal structure and function. Finally, our SIRT3 activator and inhibitor were administered systemically, which might cause some additional effects in other organs besides the CNS. It would be beneficial to modulate SIRT3 in specific brain regions in animal models to verify our results. Our study of the effects of NAM were examined in vitro and in an animal model, not clinically, so further clinical investigations of the therapeutic potential of NAD^+^/SIRT3 on cognitive impairment associated with schizophrenia are now required.

## Conclusions

In conclusion, we demonstrated that NAM could rescue neuronal apoptosis both directly and indirectly, and the SIRT3 signaling pathway might be an critical mechanism by which NAM modulates neuroinflammation and contributes to neuronal protection. These findings provide insights into the pathogenesis of schizophrenia and may help with the development of novel clinical treatment strategies.

## Supplementary information

Supplementary 1

Supplementary 2

Supplementary figures legend

## Data Availability

Data will be made available on request.
