# Supplementary figures and images for: Nicotinamide ameliorates mitochondria-related neuronal apoptosis and cognitive impairment via the NAD+/SIRT3 pathway
Source: Schizophrenia (Heidelb). 2023 May 20;9(1):32. doi: 10.1038/s41537-023-00357-w (PMC10199898; doi:10.1038/s41537-023-00357-w)

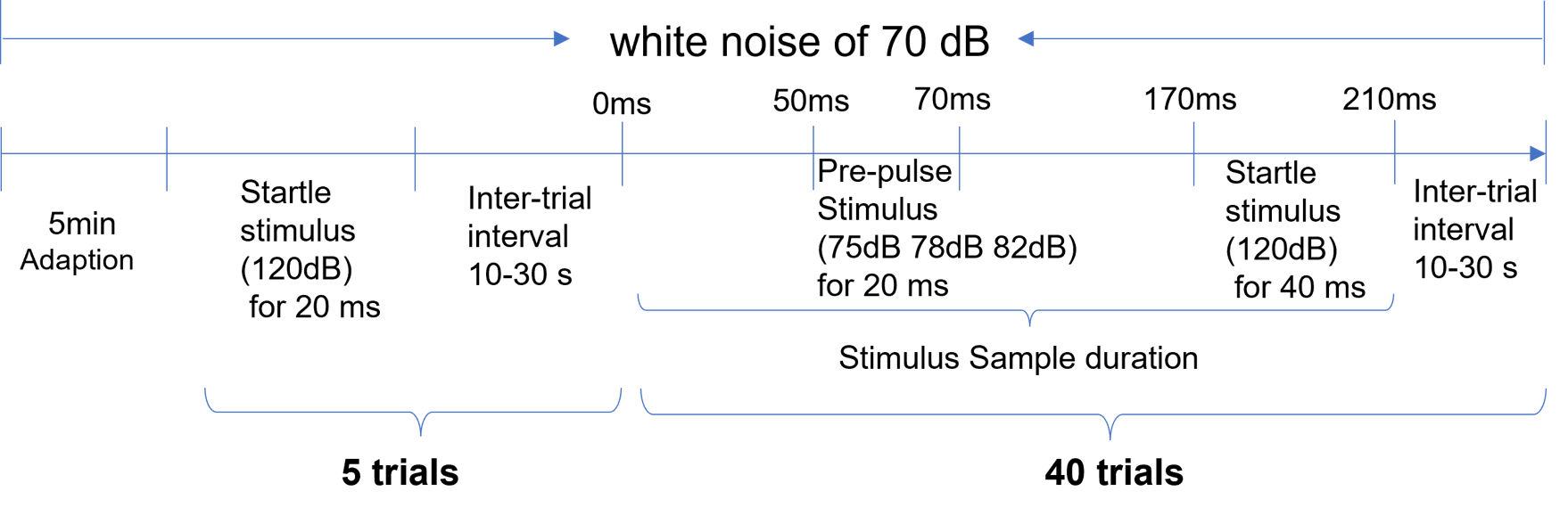

Supplement: Supplementary file 1 — Supplementary 1 [file 41537_2023_357_MOESM1_ESM.tif]

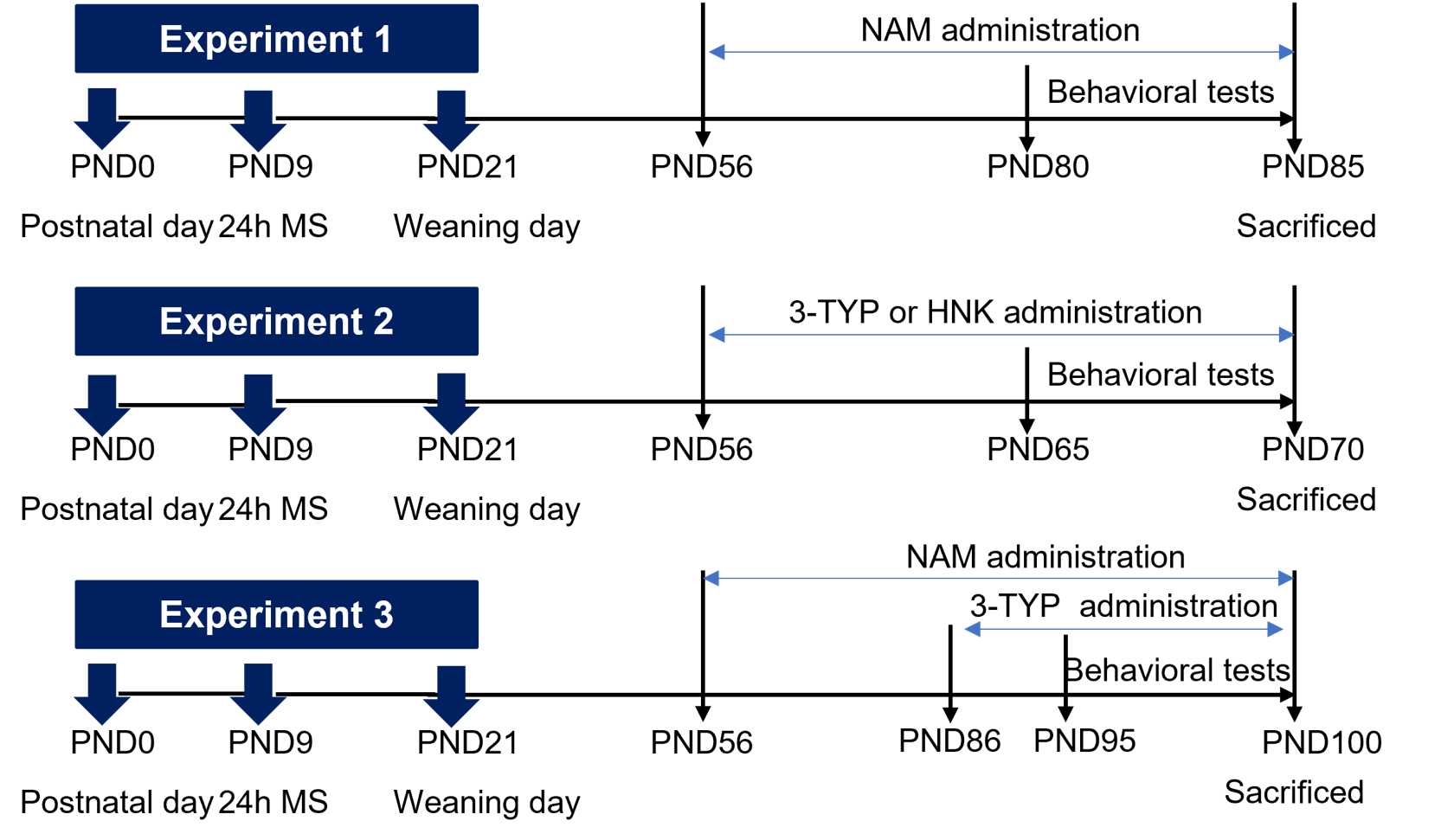

Supplement: Supplementary file 2 — Supplementary 2 [file 41537_2023_357_MOESM2_ESM.tif]
