# Supplementary material for: Nicotinamide ameliorates mitochondria-related neuronal apoptosis and cognitive impairment via the NAD+/SIRT3 pathway
Source: Schizophrenia (Heidelb). 2023 May 20;9(1):32. doi: 10.1038/s41537-023-00357-w (PMC10199898; doi:10.1038/s41537-023-00357-w)
Supplement: Supplementary file 3 — Supplementary figures legend [file 41537_2023_357_MOESM3_ESM.docx]

**Supplementary 1. Line graph depicting the stimulus protocol to assess the sensorimotor gating in PPI test.**

**Supplementary 2. Experimental program.**

Litters were subjected to MS protocols during lactation on PND9. Drug administration and behavior tests were performed on adult statement.

PND, postnatal day; MS, maternal separation.
